# The lre‐miR159a‐*LrGAMYB* pathway mediates resistance to grey mould infection in *Lilium regale*

**DOI:** 10.1111/mpp.12923

**Published:** 2020-04-21

**Authors:** Xue Gao, Qian Zhang, Yu‐Qian Zhao, Jie Yang, Heng‐Bin He, Gui‐Xia Jia

**Affiliations:** ^1^ Beijing Key Laboratory of Ornamental Plants Germplasm Innovation and Molecular Breeding, National Engineering Research Center for Floriculture, College of Landscape Architecture, Beijing Laboratory of Urban and Rural Ecological Environment Beijing Forestry University Beijing PR China; ^2^ Key Laboratory of Genetics and Breeding in Forest Trees and Ornamental Plants, Ministry of Education Beijing Forestry University Beijing PR China

**Keywords:** *Botrytis*, *GAMYB* gene, *Lilium*, miR159, plant–pathogen interaction, transgenics

## Abstract

Grey mould is one of the most determinative factors of lily growth and plays a major role in limiting lily productivity. MicroRNA159 (miR159) is a highly conserved microRNA in plants, and participates in the regulation of plant development and stress responses. Our previous studies revealed that lre‐miR159a participates in the response of *Lilium regale* to *Botrytis elliptica* according to deep sequencing analyses; however, the response mechanism remains unknown. Here, lre‐miR159a and its target *LrGAMYB* gene were isolated from *L. regale*. Transgenic *Arabidopsis* overexpressing *lre‐MIR159a* exhibited larger leaves and smaller necrotic spots on inoculation with *Botrytis* than those of wild‐type and overexpressing *LrGAMYB* plants. The *lre‐MIR159a* overexpression also led to repressed expression of two targets of miR159, *AtMYB33* and *AtMYB65*, and enhanced accumulation of hormone‐related genes, including *AtPR1*, *AtPR2*, *AtNPR1*, *AtPDF1.2*, and *AtLOX* for both the jasmonic acid and salicylic acid pathways. Moreover, lower levels of H_2_O_2_ and $O_{2}^{-}$ were observed in *lre‐MIR159a* transgenic *Arabidopsis*, which reduced the damage from reactive oxygen species accumulation. Taken together, these results indicate that lre‐miR159a positively regulates resistance to grey mould by repressing the expression of its target *LrGAMYB* gene and activating a defence response.

## Graphical abstract

lre‐miR159a positively regulates resistance to grey mould by repressing the expression of its target *LrGAMYB* gene and activating a defence response in *Lilium regale*.

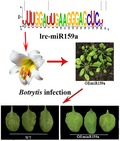

## 1 INTRODUCTION

Lily (*Lilium* spp.), as one of the most important ornamental plants in the word, can be used as both a cultivated flower crop and a potted plant. However, during both pre‐ and postharvest, especially in summer, high relative humidity provides an ideal environment for grey mould infection (Doss *et al.*, 1988; Hsieh *et al.*, 2001; van Baarlen *et al.*, 2004). Lily grey mould is caused by the fungal pathogen *Botrytis elliptica*, which has a specialized interaction with lily (Hou and Chen, 2003; Furukawa *et al.*, 2005). During the infection, spot lesions first occur on the leaves, and then the whole plant rapidly dies, causing large yield losses.

MicroRNAs (miRNAs) are small 20–24 nucleotides (nt), single‐stranded noncoding RNAs that regulate multiple biological pathways in complex organisms. Plant miRNAs mainly cleave target messenger RNAs (mRNAs) and thus regulate their expression to function in many plant biological and metabolic processes. Many plant miRNAs have been reported to respond to abiotic challenge, suggesting a broader involvement of miRNAs in defence (Jones‐Rhoades and Bartel, 2004; Phillips *et al.*, 2007; Shanfa *et al.*, 2010). For example, miR398 expression was down‐regulated by oxidative stresses, and the expression of its target genes *Cu/Zn SUPEROXIDE DISMUTASES* was increased to protect plants from oxidative damage during SO_2_ exposure (Li *et al.*, 2017). In addition, the overexpression of miR393a causes plants to become tolerant to drought stress, salt stress, and heat stress (Zhao *et al.*, 2018). Moreover, miRNAs have also been shown to be pivotal molecules in plant–pathogen interactions. For instance, miR396 was confirmed to target a set of transcription factors in the *GROWTH‐REGULATING FACTOR* (*GRF*) family in response to the necrotrophic fungi *Plectosphaerella cucumerina* and *Botrytis cinerea*, and the hemibiotrophic fungi *Fusarium oxysporum* f. sp. *conglutinans* and *Colletotrichum higginsianum*. The *MIM396* plant, in which miR396 activity is reduced, was more resistant to these fungal infections, whereas the overexpression of *MIR396* increased plant susceptibility to fungal infections (Soto‐Suárez *et al.*, 2017). Moreover, slmiR482e‐3p, which targets *FRG3* (a novel R gene) at the transcript level, is necessary for resistance to tomato wilt disease (Ji *et al.*, 2018). Several miRNAs have been reported to respond to *B. cinerea*. In tomato, miR160 and miR171a were up‐regulated, and miR169 was down‐regulated after inoculation with *B. cinerea* (Jin *et al.*, 2012). In addition, miR394 was verified as a negative regulator of *Arabidopsis* resistance to *B. cinerea* by targeting *LEAF CURLING RESPONSIVENESS* (*LCR*) (Tian *et al*., 2018). Furthermore, large numbers of miRNAs were identified in response to grey mould in *Solanum lycopersicum*, *Paeonia lactiflora*, and *Lilium regale* through high‐throughput sequencing (Jin and Wu, 2015; Zhao *et al.*, 2015; Gao *et al*., 2017), which might be associated with resistance to *Botrytis* stress.

The miR159 family is a conserved miRNA that has been found in most land plants except bryophytes (Allen *et al.*, 2007). miR159 has been found to negatively regulate *GAMYB* or *GAMYB‐like* transcription factors, which activate gibberellin (GA)‐responsive genes (Achard *et al.*, 2004). In *Arabidopsis*, seven of the miR159 targets encode proteins similar to *GAMYB*, all of which share conserved putative miR159‐binding sites of analogous complementarity, such as *MYB33*, *MYB65*, and *MYB101* (Millar, 2005; Zheng *et al.*, 2017). The functional role of the miR159‐*MYB* pathway has been analysed during seed germination floral development (Tsuji *et al.*, 2006), and primary root growth (Xue *et al.*, 2017). Despite this, more research has focused on the function of miR159 in response to multiple environmental stresses. In sugarcane, a progressive increase in miR159 transcripts was observed under short‐term polyethylene glycol stress with a concomitant down‐regulation of target *MYB* genes (Patade and Suprasanna, 2010). Similarly, root endophytic fungi induced the accumulation of miR159, enhancing the tolerance of drought stress in rice (Ehsan *et al.*, 2017). In wheat, miR159 was up‐regulated in leaves when challenged with *Puccinia striiformis* f. sp. *tritici*, resulting in a resistant phenotype through the regulation of *TaMYB3* expression (Feng *et al.*, 2013). The overexpression of miR159 caused increased resistance to powdery mildew in *Arabidopsis* (Sun, 2014). All of these indicate that miR159 is a potential positive regulator in plant response to a variety of biotic and abiotic stresses.

Previously, we systematically investigated the role of different miRNAs in the response of *L. regale* to *B. elliptica* and found that the miR159 family was significantly expressed after inoculation with *B. elliptica* (Gao *et al.*, 2017). To further our knowledge about the functions of miR159, unravelling its role in response to *Botrytis* and the underlying molecular mechanisms, we carried out this study. The miR159a and target *LrGAMYB* genes were identified and characterized in *L. regale,* and transgenic *Arabidopsis* plants overexpressing *lre‐pre‐MIR159a* were generated. Particularly interesting effects were observed in transgenic *Arabidopsis* plants overexpressing miR159a, which had markedly increased resistance to grey mould in comparison with wild‐type controls. The target of lre‐miR159a, *LrGAMYB*, is repressed in the transgenic plants. Our results reveal that the miR159a acts as a positive regulator of grey mould tolerance in *Lilium*.

## 2 RESULTS

### 2.1 Identification of lre‐miR159a in *L. regale*

**Figure 1:**
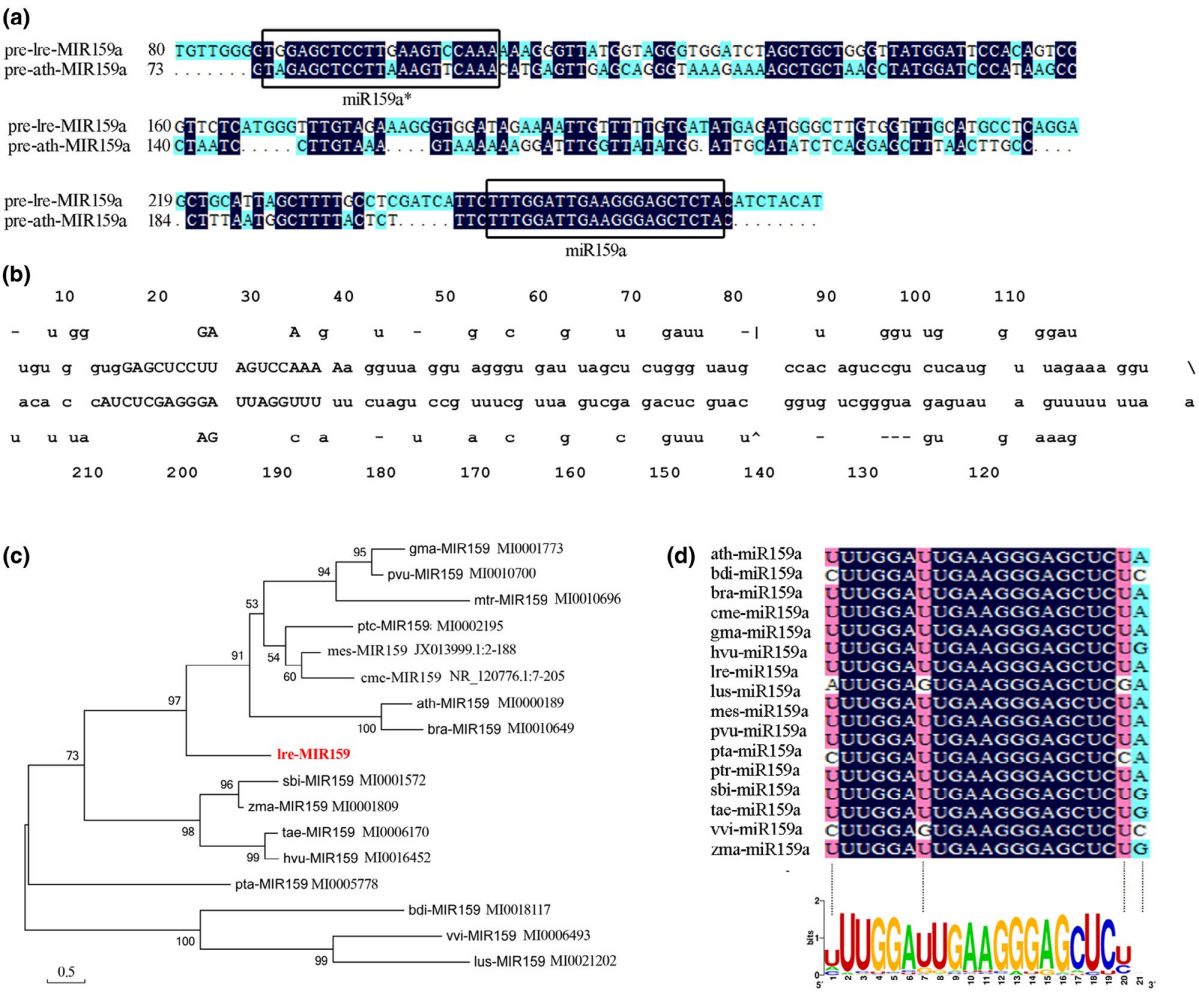
Mature and primary sequence analysis of miR159a in *Lilium regale*. (a) Sequence alignment of *pre‐lre‐MIR159a* and *pre‐ath‐MIR159a*. Boxes indicate the sequences of mature miR159a and miR159a*. (b) Proposed secondary structures for *lre‐MIR159a* precursors. (c) Phylogenetic tree of miR159a precursors in 17 species. (d) Sequence logo view of the mature miR159a sequence based on 106 miR159a sequences

We took advantage of *L. regale*, which shows resistance to grey mould, to clone lre‐miR159a. The length of precursor *lre‐MIR159a* was 219 nt, containing a 21 nt mature miR159a sequence at the 3′ end. Sequence alignment of the *MIR159a* precursor revealed 52.97% identity between *Arabidopsis thaliana* and *L. regale*, as shown in Figure 1a. A characteristic stem‐loop structure of *pre‐lre‐MIR159a* was predicted; this structure folded into a typical secondary structure (Figure 1b), with −92.40 kal/mol negative minimal folding free energies. The mature miRNA of the precursor was located in the identical stem arm (Figure 1b). This implied that the *pre‐lre‐MIR159a* could be processed correctly to form mature lre‐miR159a.

Based on the stem‐loop sequences of the *MIR159a* precursors, a phylogenetic tree was conducted with 17 species, including *L. regale* and other species, as shown in Figure 1c. Two branches were observed in the phylogenetic tree: one branch consisted of *bdi‐MIR159a*, *vvi‐MIR159a*, and *lus‐MIR159a*, while the other branch consisted of the remaining members. *MIR159a* precursors were scattered among the different species in the phylogenetic tree without clustering of monocotyledonous plants, indicating the diversity of *MIR159* sources. *lre‐MIR159a* formed an independent clade, suggesting a difference in *MIR159a* precursors from other species.

The mature miR159 sequence base conservation was analysed among 106 members, as shown in Figure 1d. The consensus mature miR159 sequence was 5′‐UUUGGAUUGAAGGGAGCUCUA‐3′ and shared high identity from the second to the 20th nucleotide. Mature lre‐miR159a shared the same sequence as the consensus sequence, indicating that lre‐miR159a might play a similar role as in other species.

### 2.2 lre‐miR159a response to *B. elliptica* infection

**Figure 2:**
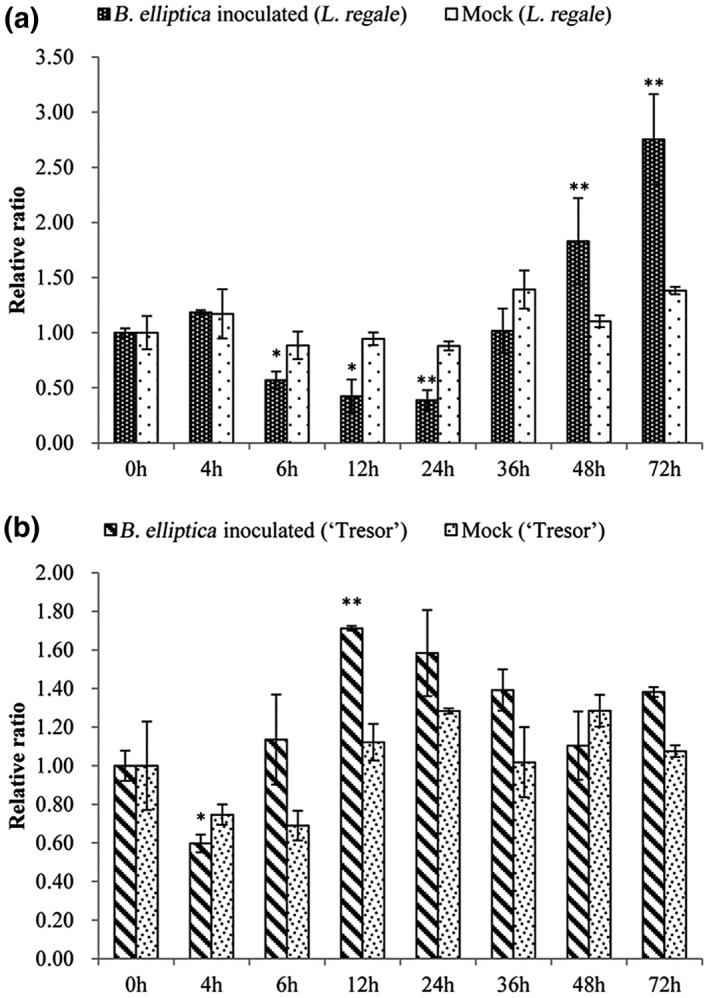
Quantitative reverse transcription PCR validations of *Botrytis elliptica*‐responsive miRNAs in the resistant *Lilium regale* and the susceptible *Lilium* cultivar Tresor. The level of expression was normalized to the level of *18S rRNA*. The normalized miRNA levels at 0 hr were arbitrarily set to 1. Each bar shows the mean ± *SE* of triplicate assays. * or ** indicates a statistically significant difference as relative to the value at 0 hr for each miRNA at *p* < .05 or .01, respectively

To elucidate the potential function of lre‐miR159a in the response to *B. elliptica* infection, the expression levels were detected by quantitative reverse transcription PCR (RT‐qPCR) among different *B. elliptica* infection times in the *B. elliptica*‐resistant *L. regale* and the *B. elliptica*‐susceptible *Lilium* Asiatic hybrid cultivar Tresor, the mock‐infected as control (Figure 2). The abundance of lre‐miR159a did not significantly change in the mock‐infected leaves at different time points. Compared with the expression level of lre‐miR159a in the control, the expression level of lre‐miR159a in resistant lines decreased before 24 hr post‐inoculation (hpi), and then a significantly up‐regulated expression pattern was exhibited following *B. elliptica* infection in resistant lilies, reaching a maximum of 2.7 times higher (Figure 2a). However, lre‐miR159a was slightly changed in susceptible Tresor during the infection process, except for a significant increase at 12 hpi. Furthermore, the levels of lre‐miR159a were significantly higher in resistant *Lilium* than in susceptible *Lilium*. The dynamic expression of lre‐miR159a in resistant *Lilium* during *B. elliptica* infection showed that lre‐miR159a was responding to *B. elliptica*, indicating that lre‐miR159a may have a role in resistance to grey mould.

### 2.3 *LrGAMYB* is targeted by miR159a through cleavage

**Figure 3:**
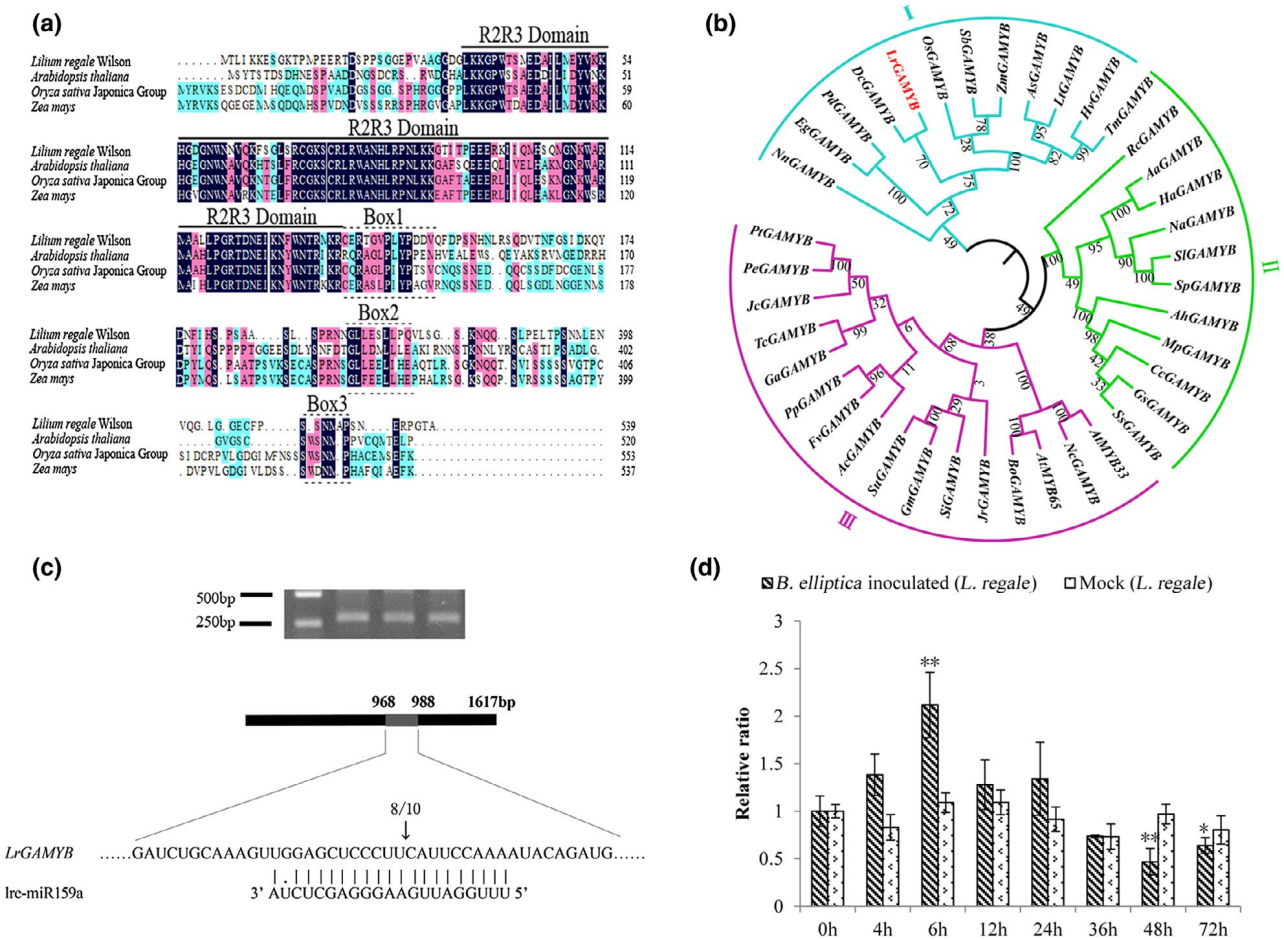
Identification and multiple sequence analysis of the miR159‐targeted *LrGAMYB* gene. (a) Amino acid alignment of the LrGAMYB protein with MYB homologues in *Arabidopsis thaliana*, *Oryza sativa* Japonica group, and *Zea mays*. (b) The phylogenetic tree of MYBs of *Lilium regale* and 37 species was constructed using the maximum‐likelihood method. (c) The RNA ligase‐mediated rapid amplification of cDNA ends (RLM‐RACE) products for the predicted *LrGAMYB* gene amplified and positions of the cleavage. Mapping of lre‐miR159a‐guided cleavage sites in *LrGAMYB* mRNA by RLM‐RACE. Vertical arrow indicates the 5′ position of the cleaved mRNA fragment, and the number indicates the number of independent clones analysed in the different tissues. (d) Quantitative reverse transcription PCR analysis of *LrGAMYB* in *L. regale* after *Botrytis elliptica* inoculation

It is well known that plant miRNAs function through cleaving target genes. Therefore, identifying target genes of mRNAs is important to understand their specific contributions. In plants, miR159 was predicted to target MYBs with a strongly conserved miR159‐binding site. To identify miR159 target genes in *Lilium*, psRNATarget was used to predict target genes based on the *Lilium* transcriptome, and one cDNA fragment, characterized as *GAMYB*, was selected for further analysis. Through rapid amplification of cDNA ends (RACE), the full‐length cDNA of the *LrGAMYB* gene was isolated, which contains a 1617 nucleotide (nt) open reading frame and encodes a 539 amino acid polypeptide. Sequence alignment of the LrGAMYB protein sequence with *A. thaliana*, *Oryza sativa* Japonica group and *Zea mays* sequences is shown in Figure 3a. Similar to other species, the LrGAMYB protein possesses an R2R3 DNA‐binding domain in the N‐terminus and three conserved motifs (Box1, Box2, and Box3). In addition, the LrGAMYB protein sequence shares high similarity with GAMYB in monocots, such as *Elaeis guineensis* (49%) and *Phoenix dactylifera* (48%).

A maximum‐likelihood phylogenetic tree was established with the LrGAMYB protein sequences and 37 homologues from several species (Figure 3b). The evolutionary tree of related genes was divided into three parts (I, II, and III). LrGAMYB formed a clade with DcGAMYB, which belongs to group I with members mainly from monocots (Figure 3b). Furthermore, the *LrGAMYB* gene exhibited base pairing with near‐perfect complementarity with lre‐miR159a, suggesting *LrGAMYB* was the putative target of lre‐miR159a.

To confirm whether *LrGAMYB* genes are direct targets of lre‐miR159a, a modified 5′ RNA ligase‐mediated rapid amplification of cDNA ends (RLM‐RACE) was conducted to examine the miR159‐directed cleavage sites of *LrGAMYB* transcripts. Generated cleavage products were amplified and cloned into a vector. The 5′ end sequencing of the amplified products was sequenced in independent clones, suggesting that cleavage sites are located in the middle of the lre‐miR159a complementary region. Cleavage occurred at an identical position, which corresponded to the 10th nucleotide position of the consensus mature miR159a sequence, in 8 of the 10 tested samples (Figure 3c). Furthermore, *L. regale* plants were inoculated with *B. elliptica* to investigate the expression pattern of *LrGAMYB* using RT‐qPCR analysis (Figure 3d). This revealed that *LrGAMYB* was up‐regulated in the initial *B. elliptica* infection and significantly reduced at later times. The expression level of *LrGAMYB* was negatively correlated with lre‐miR159a in *L. regale*. These results confirm that *LrGAMYB* is an authentic target of lre‐miR159a, and *LrGAMYB* is subject to miR159‐mediated down‐regulation.

### 2.4 lre‐miR159a positively regulates plant resistance to *B. elliptica*

**Figure 4:**
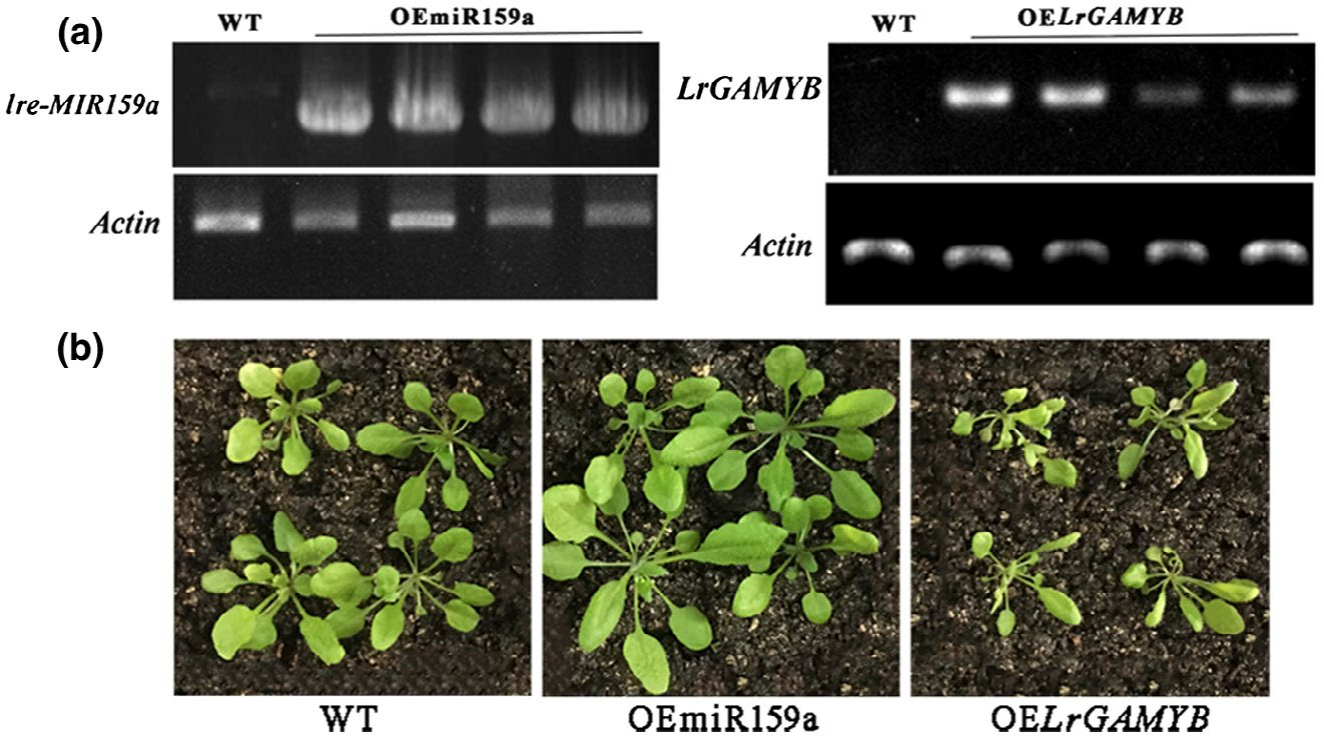
Phenotypic characteristics of wild‐type (WT), transgenic *Arabidopsis* plants overexpressing *lre‐MIR159a* (OEmiR159a), and overexpressing *LrGAMYB* (OE*LrGAMYB*) under the control of the 35S promoter (35S:*lre‐MIR159a* and 35S:*LrGAMYB*). (a) Agarose gel analysis of lre‐miR159a and *LrGAMYB* genes in transgenic *Arabidopsis*. (b) Comparison of 4‐week‐old plants between transgenic lines and WT

To study the function of lre‐miR159a and *LrGAMYB* in plant pathogens, we constructed two plasmids overexpressing lre‐miR159a and *LrGAMYB*, respectively, and transformed these into the *Arabidopsis* Col‐0 plants, which was used as the wild type (WT). The stem‐loop precursor of *lre‐MIR159a* and *LrGAMYB* were under the control of the CaMV 35S promoter and linked to pCAMBIA1301 vector (35S:*lre‐MIR159a* and 35S:*LrGAMYB*). Multiple transgenic lines were identified through *lre‐MIR159a* and *LrGAMYB* amplification (Figure 4a). It seems that lre‐miR159a meditates plant development, resulting in increased plant size after 4 weeks of growth (Figure 4b). In addition, *Arabidopsis* overexpressing miR159a (OEmiR159a) was evaluated for stem elongation and early flowering. However, *Arabidopsis* plants overexpressing *LrGAMYB* (OE*LrGAMYB*) exhibited short stature along with reduction in stem diameter and length of leaves.

**Figure 5:**
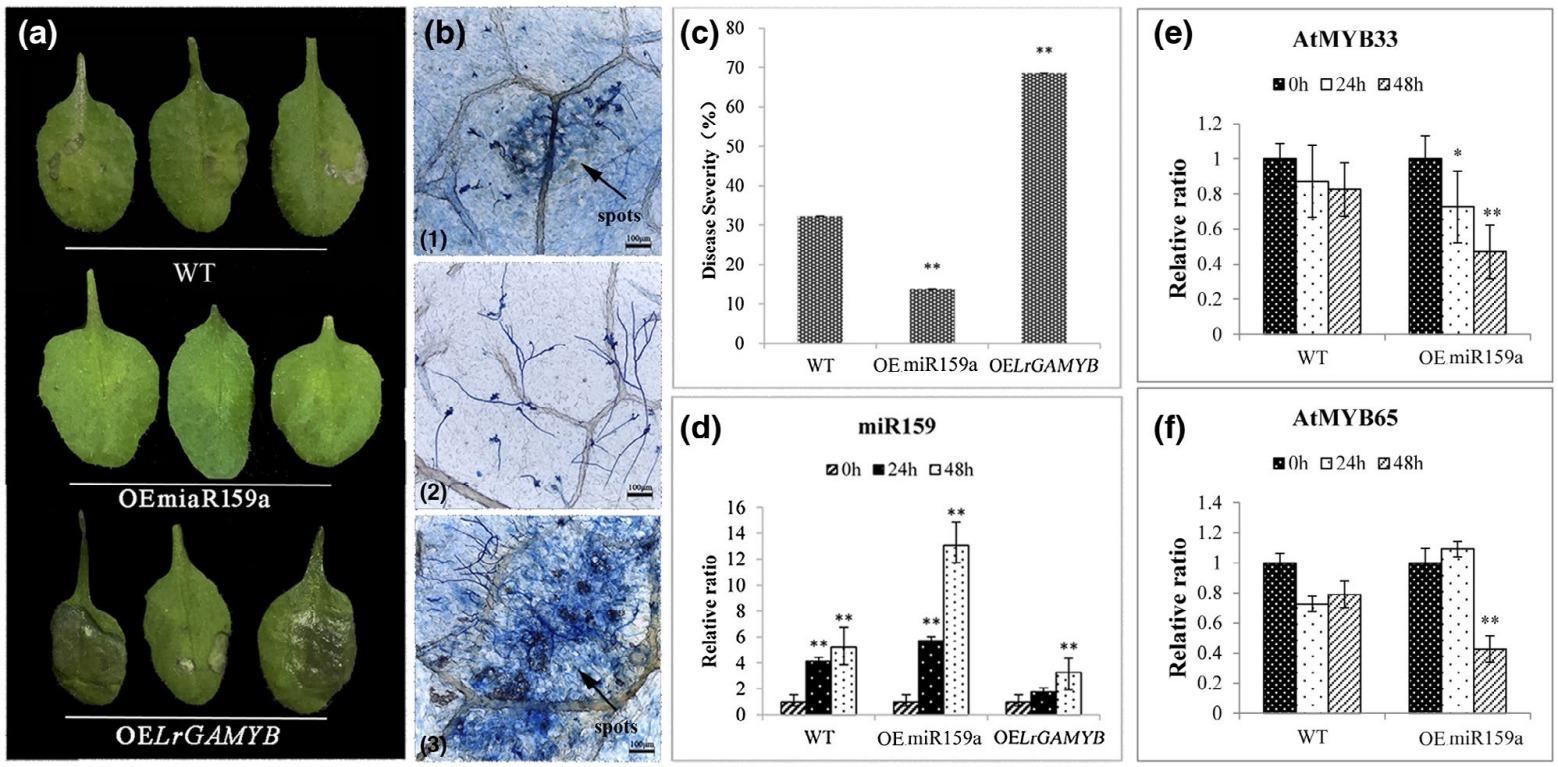
Plant disease assay following *Botrytis cinerea* inoculation. (a) Disease symptoms in *Arabidopsis* wild‐type (WT), overexpressing line OEmiR159a, and overexpressing line OE*LrGAMYB*. Leaves were collected at 2 days after *Botrytis* inoculation. Similar results were observed in three to five replicate experiments. (b) Trypan blue staining of infected leaves after *B. cinerea* inoculation. (1) to (3) represent WT, OEmiR159a, and OE*LrGAMYB Arabidopsis*, respectively. Scale bar = 100 μm. (c) Disease severity of WT, OEmiR159a, and OE*LrGAMYB Arabidopsis*; (d–f) Relative expression levels of miR159a and miR159a‐targets, *AtMYB33*, and *AtMYB65*, in WT and OEmiR159a *Arabidopsis* after *B. cinerea* inoculation

To determine whether lre‐miR159a and *LrGAMYB* play a role in the response to *Botrytis*, both WT and overexpression plants were inoculated with discs of *B. cinerea*, and the disease progression was observed 2 days post‐inoculation (dpi). Minimal growth of *Botrytis* was observed on the leaf surface of the OEmiR159a, and water‐soaked symptoms appeared. However, infected lesions and necrosis were observed on WT and OE*LrGAMYB* (Figure 5a). Necrosis reached nearly 70% on the leaves of OE*LrGAMYB Arabidopsis* at 48 hpi (Figure 5c). Spores of *B. cinerea* were also used for the inoculation, and the results are shown in Figure S1, which were same for mycelial (disc) infection. Trypan blue staining further verified these results. The proliferation of the fungal mycelia was widespread in OE*LrGAMYB*, accompanied by the development of spots and necrosis (Figure 5b). Fewer hyphae and lesions were observed in OEmiR159a, suggesting lre‐miR159a positively regulates resistance to grey mould.

Moreover, RT‐qPCR was used to verify the expression of lre‐miR159a in the WT and transgenic plants (OEmiR159a and OE*LrGAMYB Arabidopsis*). miR159a was up‐regulated during the *Botrytis* infection process; however, the miR159a levels in the OEmiR159a lines were significantly higher than those in WT and OE*LrGAMYB Arabidopsis* (Figure 5d). These results suggest that lre‐miR159a is successfully expressed in transgenic *Arabidopsis* and that overexpression could enhance the resistance to pathogen infection. Additionally, we further studied the expression of the miR159a‐targeted genes in *Botrytis*‐inoculated OEmiR159a *Arabidopsis* (Figure 5e,f). The miR159a‐targeted genes *AtMYB33* and *AtMYB65* were down‐regulated in OEmiR159a *Arabidopsis* during the pathogen infection process, and this expression pattern contrasted with miR159a, suggesting miR15a regulates the target *GAMYB* gene in response to *Botrytis* infection.

### 2.5 The expression of hormone‐related genes in lre‐miR159a overexpression *Arabidopsis* after infection with *B. cinerea*

**Figure 6:**
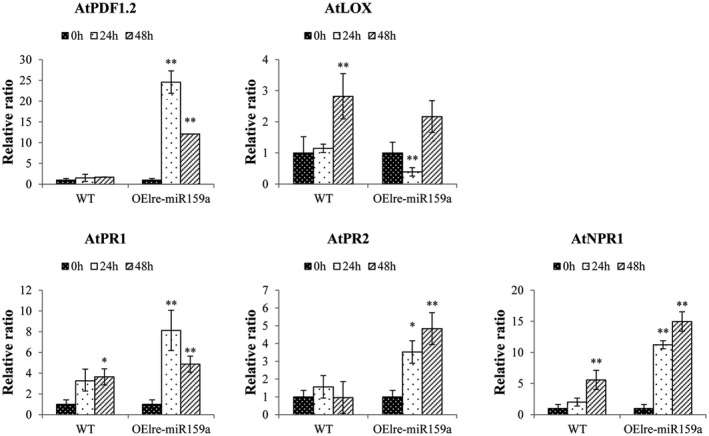
The expression of hormone‐related genes of wild‐type (WT) and *Arabidopsis* overexpressing miR159a (OElre‐miR159a) upon inoculation with *Botrytis cinerea*. The level of expression was normalized to the level of the *AtActin* gene. The normalized gene levels at 0 hr were arbitrarily set to 1. Each bar shows the mean ± *SE* of triplicate assays. * or ** indicates a statistically significant difference relative to the value at 0 hr for each gene at *p* < .05 or .01, respectively

Phytohormone signalling pathways, such as salicylic acid (SA) and jasmonic acid (JA), are involved in the control of the initiation of defence mechanisms against *B. cinerea* (Zhao *et al*., 2003; Blanco‐Ulate *et al.*, 2013). To investigate the possible signalling pathways involved in miR159‐mediated resistance, the transcript levels of genes in the SA‐ and JA‐dependent pathways during fungal inoculation were detected. Both the SA‐responsive marker genes (*AtNPR1*, *AtPR1*, and *AtPR2*) and the JA‐responsive marker gene (*AtPDF1.2*) were significantly expressed higher in *Botrytis*‐inoculated OEmiR159a *Arabidopsis* (Figure 6) than in WT, which indicates that the OEmiR159a plants might mediate grey mould resistance by activating the SA and JA signalling pathways. The transcription of *AtLOX* in JA pathway was down‐regulated, suggesting that JA might play a role in later infection processes.

### 2.6 miR159a overexpression balances ROS homeostasis and increased resistance to *Botrytis*

**Figure 7:**
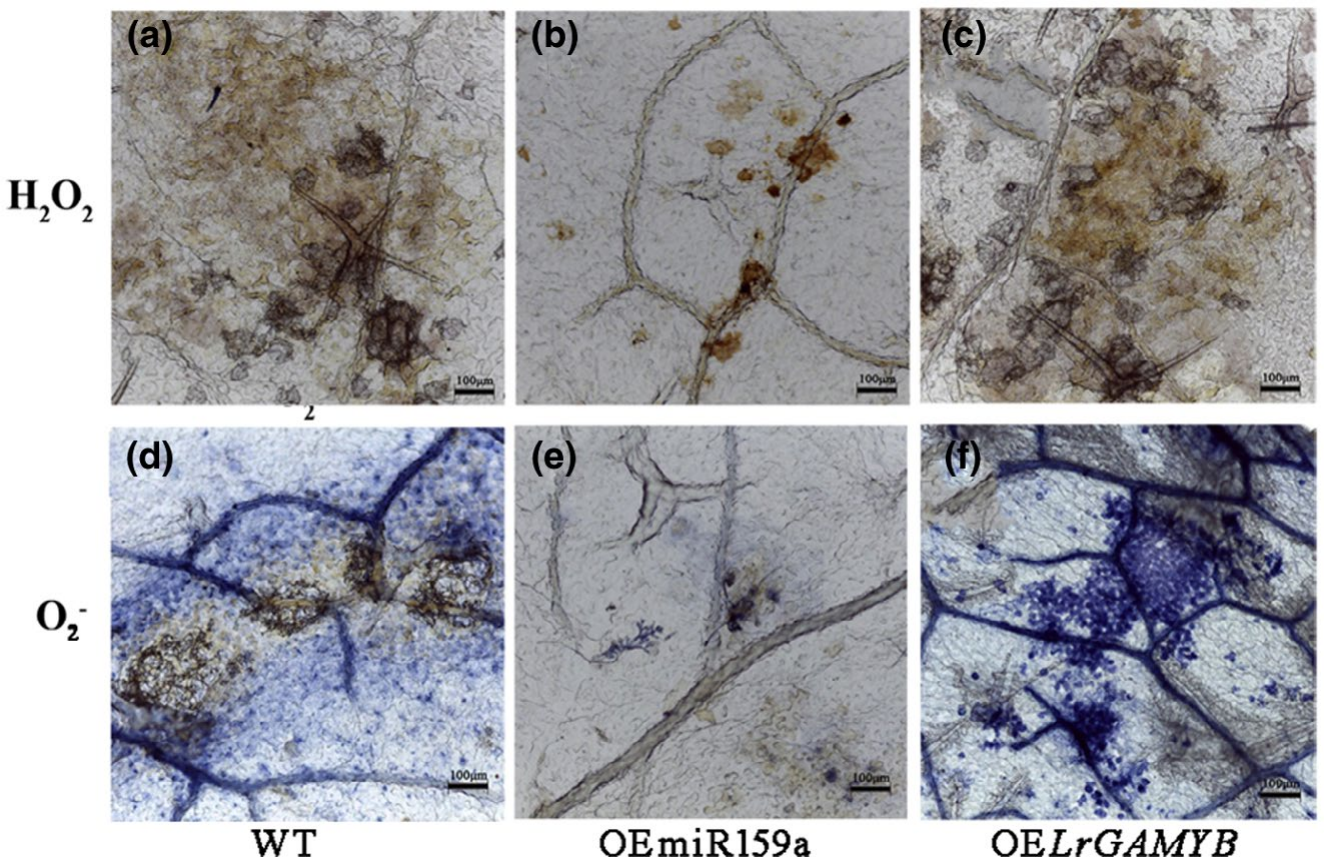
Temporal evolution of H_2_O_2_ and $O_{2}^{-}$ accumulation in the leaves of wild‐type (WT) and transgenic *Arabidopsis* overexpressing miR359a (OEmiR359a) or *LrGAMYB* (OE*LrGAMYB*) following *Botrytis cinerea* inoculation for 48 hr. Scale bar = 100 μm. (a) to (c) are stained with 3,3′‐diaminobenzidine and show the accumulation of H_2_O_2_; (d) to (f) are stained with nitroblue tetrazolium and show the accumulation of $O_{2}^{-}$

Because one of the earliest defence responses in *B. cinerea* is reactive oxygen species (ROS) production (Asselbergh *et al.*, 2007; van Kan, 2006), we monitored H_2_O_2_ and $O_{2}^{-}$ level fluctuations when WT and transgenic *Arabidopsis* were inoculated with *B. cinerea*. After inoculation with *B. cinerea* for 2 days, the transgenic and WT *Arabidopsis* both had increased H_2_O_2_ levels, and the brown colour of OE*LrGAMYB* leaves was deeper than that of WT leaves and OEmiR159a leaves (Figure 7a–c), suggesting that OE*LrGAMYB* plants had higher ROS levels than WT and OEmiR159a *Arabidopsis*. The excess H_2_O_2_ generation was apparent in susceptible OE*LrGAMYB* transgenic *Arabidopsis*, suggesting that infection accelerated H_2_O_2_ accumulation, resulting in cellular damage.

Similarly, the bluish violet staining was reduced in OEmiR159a transgenic *Arabidopsis* but widespread in the intercellular spaces and epidermal cells of WT and OE*LrGAMYB* transgenic *Arabidopsis* leaves after inoculation (Figure 7d–f). $O_{2}^{-}$ accumulation expanded from the sites of fungal contact, where rot spots were apparent. These data indicate that altering miR159a expression enhanced H_2_O_2_ and $O_{2}^{-}$ homeostasis, which may ultimately increase resistance to *Botrytis*.

## 3 DISCUSSION

The miR159 family is one of the ancient miRNA families in not only in monocotyledons and dicotyledons but also gymnosperms and ferns, such as *Picea abies* (Xia *et al.*, 2015), *Pinus taeda* (Lu *et al.*, 2007), and *Selaginella moellendorffii* (Axtell *et al.*, 2007). Furthermore, the miR159 family differs in the number of mature and precursor genes among species. In *Arabidopsis*, this family is encoded by three genes and forms three mature miR159 members (miR159a, miR159b, and miR159c) located in different regions of the genome (Allen *et al.*, 2007). Further research has indicated that a mir159a mir159b double mutant has pleiotropic morphological defects, including altered growth habits, curled leaves, small siliques, and small seeds. Neither mir159a nor mir159b single mutants displayed any of these traits, indicating functional redundancy (Allen *et al.*, 2007; Alonso‐Peral *et al.*, 2010). In our previous research, five miR159 members were identified in *L. regale* through high‐throughput sequencing; however, lre‐miR159a was significantly more highly and differentially expressed than others members after inoculation with *B. elliptica*, suggesting that lre‐miR159a might respond to *B. elliptica* in *L. regale* (Gao *et al.*, 2017). Therefore, we chose lre‐miR159a for further research in this study.

The results of sequence alignment analysis suggested that mature miR159a sequences are highly conserved in different species; however, the precursors have substantial differences. For instance, *Z*. *mays* and *P*. *abies* possess the most miR159 precursors with 11 genes, but 11 species hold only one. Furthermore, the phylogenetic tree showed that the evolutionary distance separating the branches of different species of miR159a precursors is long, and the sequence similarity is relatively low, suggesting varied sources of mature miR159a. *pre‐lre‐MI159a* formed an independent clade, suggesting it is different from other species. The highly conserved sequences of mature miR159 indicate that they may perform similar functions.

Studies have revealed that miR159 plays an important role in the response to stresses. For instance, miR159 responds to salinity stress (Kitazumi *et al.*, 2015), drought stress (Mohsenifard *et al.*, 2017), and heat stress (Li *et al.*, 2016). Suppression of miR159 in plants resulted in short stature along with smaller stem, leaf, and grain size, which enhanced the hypersusceptibility to adverse environmental stress (Zhao *et al.*, 2017). Furthermore, altered accumulation of miR159a levels was observed during tomato leaf curl virus infection in tomato, indicating that miR159 behaves as an active factor in pathogen resistance (Koundal *et al.*, 2010). In our previous study, we also found by high‐throughput sequencing that lre‐miR159a was involved in the response to *Botrytis*. miR159 mainly contributes to the regulation of plant development and stress by targeting *MYB* transcription factors (Millar, 2005; Xue *et al.*, 2017). The *GAMYB* or *GAMYB‐like* genes encode a highly conserved family of R2R3 MYB domain transcription factors implicated in GA signal transduction (Woodger *et al.*, 2003). Studies have indicated that the *GAMYB* transcription factor superfamily is involved in plant development and metabolism (Alonso‐Peral *et al.*, 2010; Sheldon *et al.*, 2001) and response to pathogens and abiotic stress (Yang *et al*., 2014; Butt *et al.*, 2017). Moreover, studies have revealed that *MYB* transcriptome factors participate in response to *B. cinerea*. For instance, the overexpression of *AtMYB44* in *Arabidopsis* results in a stronger ROS burst, greater cell death, and severe necrosis symptoms, which enhances susceptibility to *B. cinerea* (Shi *et al.*, 2011). Furthermore, *MYB46*, thought to regulate secondary cell wall biosynthesis in the vascular tissue of the stem, functions as a disease susceptibility modulator to *B. cinerea*. The *MYB46‐*mutant plants exhibited increased disease resistance to *B. cinerea* (Vicente *et al.*, 2011). These results suggest that *MYB* transcription factors are negative regulators in response to *B. cinerea*. In this study, *LrGAMYB* transcription factors were verified as one target of lre‐miR159a in *L. regale* by RLM‐RACE. After inoculation with *B. elliptica*, lre‐miR159a was significantly up‐regulated in resistant *Lilium*, accompanied by decreased expression of *LrGAMYB*, indicating a negative relationship between lre‐miR159a and its target *LrGAMYB*. These results reveal that lre‐miR159a and its target *LrGAMYB* responded to *Botrytis* infection in lily.

To further study the role of lre‐miR159a and the target *LrGAMYB* gene in disease resistance, transgenic *Arabidopsis* of the lre‐miR159a and *LrGAMYB* genes were constructed. The OEmiR159a plants exhibited larger leaves and smaller necrotic spots than the WT and OE*LrGAMYB Arabidopsis* plants after *Botrytis* inoculation (Figure 5). The results indicate that the molecular and physiological responses to *Botrytis* included ROS production and transcriptional responses. A stronger response of JA‐ and SA‐mediated defence genes was detected in the *Botrytis*‐infected OEmiR159a plants (Figure 6), implying that overexpression of miR159a enhances transgenic plant defence by activating the hypersensitive response. An SA‐dependent signalling pathway led to the expression of pathogenesis‐related (PR) proteins, including *AtNPR1*, *AtPR1*, and *AtPR2*, thus contributing to resistance. *AtPDF1.2*, which is dependent on the JA pathway, was also induced and was highly expressed. In agreement with previous reports, the SA and JA signalling pathways interact extensively and cooperatively in response to *Botrytis* infection (Glazebrook, 2005).

*B. cinerea* is a nonspecific necrotrophic fungal pathogen that triggers plants to generate large amounts of ROS and induces local cell death to facilitate infection (Su *et al.*, 2011; Pietrowska *et al.*, 2015). Previous studies have shown that the induction of H_2_O_2_ in plant cells, accompanied by $O_{2}^{-}$ generation, can promote programmed cell death in the host and the expansion of disease lesions to facilitate *B. cinerea* infection (Govrin and Levine, 2000; Patykowski, 2006; Asai and Yoshioka, 2009; Wan *et al.*, 2015). During the early stages of *Botrytis* infection, the ROS burst can induce a defensive reaction. However, high H_2_O_2_ levels can disturb redox homeostasis, trigger initiation of cell death, and facilitate necrotrophic pathogen attack of host plants. In our study, H_2_O_2_ and $O_{2}^{-}$ levels were lower in OEmiR159a *Arabidopsis* than in WT and OE*LrGAMYB Arabidopsis*, most probably due to the overexpression of lre‐miR159a. These results indicate that lre‐miR159a plays a positive role in resistance to *Botrytis* by suppressing the target *LrGAMYB* gene.

We also found an interesting phenomenon in which OEmiR159a *Arabidopsis* showed early flowering. Studies have indicated that miR159 is involved in floral organ development. For instance, miR159 expression regulates floral transition (Li *et al.*, 2013), flower development (Wang *et al.*, 2017), and timing of flowering (Guo *et al.*, 2017). Furthermore, miR159 is required for fruit set (da Silva *et al.*, 2017), and the accumulation of miR159 could affect seedling development. These results suggest multiple regulatory networks of miRNAs that participate in plant growth and development. Further research is needed to verify the functions of these miRNAs and their regulatory networks.

## 4 EXPERIMENTAL PROCEDURES

### 4.1 Plant materials and *B. elliptica* inoculation

Fresh, uniform‐sized bulbs of *L. regale* were held in cold storage (approximately 1 °C) for a month before planting. After this vernalization period, bulbs were planted on culture medium under a 12 hr day/night cycle at 25/22 °C and maintained in a semi‐open house covered with a shading screen at Huairou, Beijing Province, China. The fungus, *B. elliptica*, isolated from diseased lily leaves, was maintained on potato dextrose agar at 22 °C under near‐UV light in a 100% relative humidity chamber. For the bioassay, 7‐day‐old cultures were used. Conidia were collected by gently vortexing in Tween 20 and adjusted to a concentration of 5 × 10^4^ conidia/ml for inoculation.

*Arabidopsis* Columbia (Col‐0) was used for transformation by *pre‐lre‐MIR159a* and *LrGAMYB* plasmids. Seeds were sown on 1/2 × Murashige and Skoog (MS) medium, cold treated for 1 week at 4 °C, and transferred to a controlled environment incubator with a 16/8 hr light/dark photoperiod and a 25/18 °C day/night thermoperiod.

### 4.2 Sequence analysis of precursor miR159a from *L. regale* and prediction target genes

Total genomic DNA was extracted from leaves using the New Plant Genomic DNA Extraction Kit (Tiangen). The DNA sequence was amplified by PCR using primers designed based on the small‐RNA library previously constructed by our laboratory (Gao *et al.*, 2017), and the primers are shown in Table S1. PCR amplification was performed with KOD‐Plus‐Neo polymerase (Totobo) according to the instruction manual, with a programme set as follows: 94 °C for 2 min; and 45 cycles of 98 °C for 10 s, 59 °C for 15 s, and 72 °C for 15 s. The PCR products were gel purified and cloned into the pLB vector (Tiangen) and then sequenced by Sangon Biotech (Shanghai).

Mfold web server (http://unafold.rna.albany.edu/?q=mfold/RNA-Folding-Form) was used to examine the hairpin structure of the miR159a precursor. Precursors of miR159a from other plants were obtained from miRBase 21.0 and aligned with the miR159a precursor sequence by DNAMAN (Lynnon Biosoft) and ClustalW. The target genes of miR159a were predicted by psRNATarget web (http://plantgrn.noble.org/psRNATarget/) with default parameters (Dai and Zhao, 2011). The prediction of the lre‐miR159a targets was based on the transcriptome of *L. regale*, which was constructed by our laboratory. A phylogenetic tree was constructed with MEGA 6 using the neighbour‐joining method. The conserved domains of mature miR159a sequences were aligned using the Weblogo program with default parameters (http://weblogo.berkeley.edu/logo.cgi) (Crooks *et al.*, 2004).

### 4.3 Cloning the lre‐miR159a‐targeted *GAMYB* gene and sequence analysis

The lre‐miR159a‐targeted *GAMYB* partial sequence was deduced from the *L. regale* transcriptome. First, total RNA from *L. regale* leaves was extracted using EASYspin Plus RNA Kit (Aidlab). Full *GAMYB* cDNA fragments were then obtained with the SMARTer RACE 5′/3′ Kit (Takara) followed by the second round of nested PCR with specific outer and inner primers listed in Table S2. The full‐length of *GAMYB* gene sequence was amplified with designed primers (Table S1) and cloned into the pLB vector. These GAMYB protein sequences of other plants were obtained from the NCBI database (https://www.ncbi.nlm.nih.gov/). Multiple alignments of these protein sequences were aligned using DNAMAN.

### 4.4 RNA ligase‐mediated rapid amplification of cDNA ends

To detect the miRNA‐target cleavage site, RLM‐RACE was conducted using the 5′‐Full RACE Kit (Takara) with a 5′‐RACE adaptor (Lu *et al.*, 2017). Total RNA for RLM‐RACE was obtained from *L. regale* leaves. The 5′‐RACE outer primer and gene‐specific outer primer (GSP1) were used for the first round of nested PCR, followed by the second round of nested PCR using the 5′‐RACE inner primer and gene‐specific inner primer (GSP2) (Table S2). Amplification products were gel purified, cloned into the pRACE vector, and sequenced.

### 4.5 Plasmid construction and *Arabidopsis* transformation

To overexpress miR159a and *LrGAMYB*, the sequence of the precursor lre‐miR159a and full cDNA fragments encoding *LrGAMYB* were amplified from the corresponding cloned vectors and then inserted downstream of the CaMV 35S promoter in pCAMBIA1301 (GenBank no. AF234297). The recombinant vectors were then introduced into *Agrobacterium tumefaciens* GV3101 and the *Arabidopsis* Col‐0 WT was used for transformation by the floral‐dip method. Transformants were selected with 50 mg/L hygromycin B and confirmed by reverse transcription PCR.

Three homozygous T_3_ lines were established and used for the treatments. For the bioassay, discs of *B. cinerea* mycelia and conidia solution were both used for the inoculation of detached leaves. Discs of mycelia were punched from the growing edge of colonies, and conidia were adjusted to a concentration of 5 × 10^5^ conidia/ml for inoculation. Trypan blue staining for the presence of the fungal infection was performed as previously described (Gao *et al.*, 2018). After the decolorization treatment, the mycelium in tissue was observed with a microscope (BME, Leica).

### 4.6 RNA insolation and RT‐qPCR

Total RNAs were extracted and reverse transcribed using the First Strand cDNA Synthesis Kit (Toyobo). RT‐qPCR was conducted in a total volume of 20 μl containing 10 μl SYBR Premix Ex Taq (Takara) using following programme: 5 min denaturation at 94 °C; followed by 30 cycles of 94 °C for 5 s, 60 °C for 20 s, and 72 °C for 20 s. The *18S rRNA* and *CLATHRIN* genes were used as the reference genes for normalization in *L. regale*, and the *AtActin* gene was used for *A. thaliana*. Each sample was performed in triplicate and the mean value of technical replicates was recorded for each biological replicate. The primers used are listed in Table S3.

For RT‐qPCR analysis of lre‐miR159a and *LrGAMYB* to grey mould response in *Lilium*, leaves inoculated with *B. elliptica* were harvested at 0, 2, 6, 12, 24, 36, 48, and 72 hpi of the *B. elliptica*‐resistant *L. regale* and the *B. elliptica*‐susceptible cv. Tresor according to previous studies (Gao *et al.*, 2018).

For RT‐qPCR analysis in *Arabidopsis*, inoculated leaves were harvested at 0, 24, and 48 hpi for RNA extraction.

### 4.7 3,3′‐diaminobenzidine and nitroblue tetrazolium staining

H_2_O_2_ and $O_{2}^{-}$ were detected by previously described 3,3′‐diaminobenzidine (DAB) and nitroblue tetrazolium (NBT) staining protocols, respectively, to compare the ROS responses of WT and transgenic *Arabidopsis*. To detect the production of H_2_O_2_, leaves were immersed in a DAB solution (1 mg/ml acidified with HCl to pH 3.8) under direct light for 2 hr after samples were inoculated with *B. cinerea* 48 hpi. The stained leaves were incubated in a solution of 70% ethanol and then photographed.

For observation of $O_{2}^{-}$, leaves were collected directly into 0.1% NBT solution in 10 mM phosphate buffer (pH 7.8) prior to vacuum infiltration for 30 min and exposure to direct light for 20 min. The NBT‐stained samples were then observed.

### 4.8 Statistical analysis

All data are presented as the mean ± *SE* and were subjected to analysis of variance according to Student's *t* test (*p < .05, **p < .01). Each data set was independently compared with the control data set to determine significant differences.

## Data Availability

The data that support the findings of this study are available from the corresponding author upon reasonable request.
