# Supplementary figures and images for: The lre‐miR159a‐LrGAMYB pathway mediates resistance to grey mould infection in Lilium regale
Source: Mol Plant Pathol. 2020 Apr 21;21(6):749–60. doi: 10.1111/mpp.12923 (PMC7214475; doi:10.1111/mpp.12923)

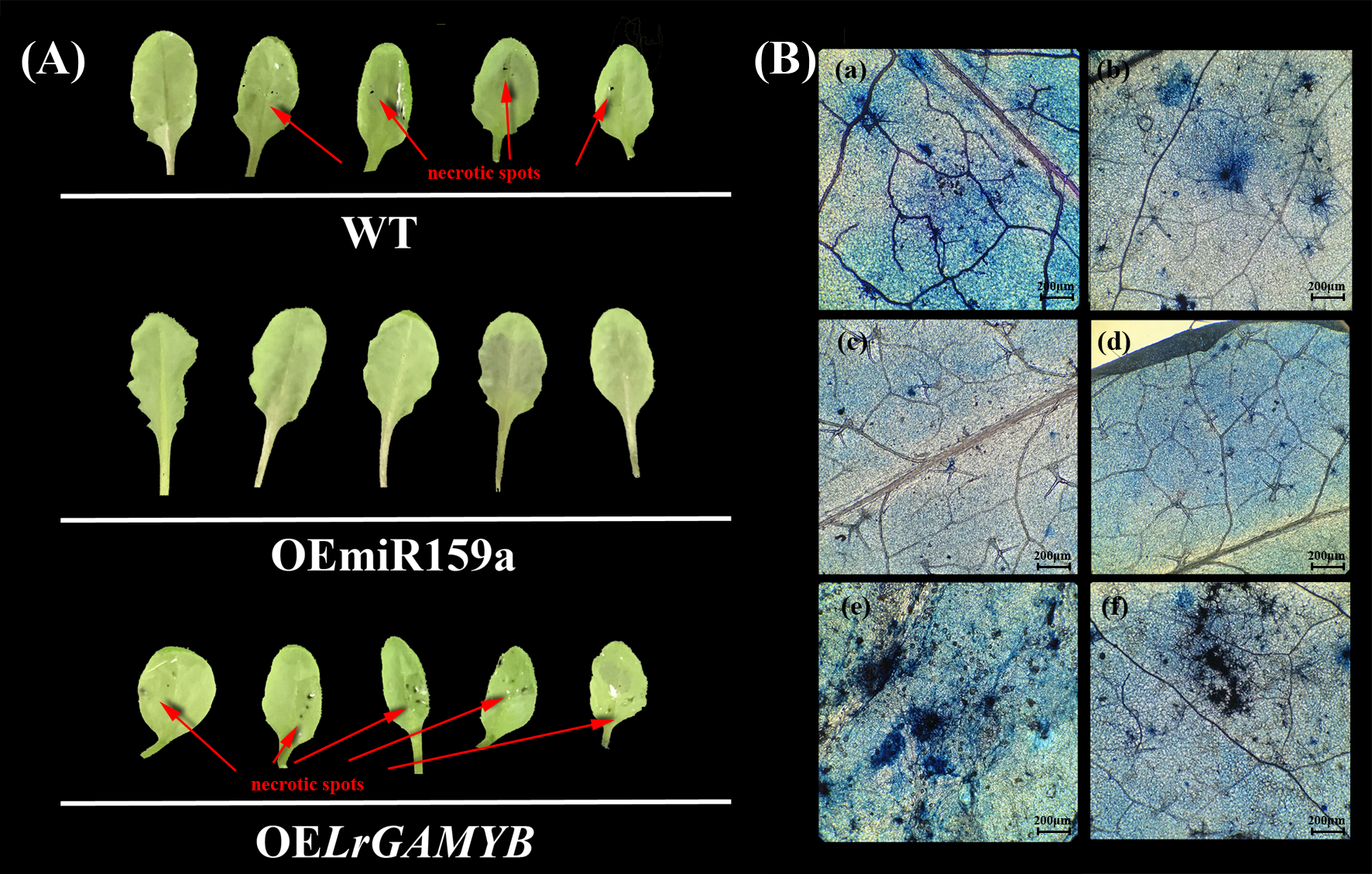

Supplement: Supplementary file 1 [file MPP-21-749-s001.tif]
